# What do nonprofit hospitals reward? An examination of CEO compensation in nonprofit hospitals

**DOI:** 10.1371/journal.pone.0264712

**Published:** 2022-03-21

**Authors:** Karen Mulligan, Seema Choksy, Catherine Ishitani, John A. Romley

**Affiliations:** 1 Sol Price School of Public Policy, University of Southern California, Los Angeles, California, United States of America; 2 Leonard D. Schaffer Center for Health Policy & Economics, University of Southern California, Los Angeles, California, United States of America; 3 School of Pharmacy, University of Southern California, Los Angeles, California, United States of America; University of Bologna, ITALY

## Abstract

Nonprofit hospital chief executive officer (CEO) compensation has received considerable attention in light of nonprofits’ tax-favored status as well as the high costs of hospital care. Past studies have found that hospital financial performance is a significant determinant of CEO pay but nonprofit performance, including quality and charity care, are not. Using post-ACA data, we re-examine whether a variety of hospital performance measures are important determinants of nonprofit hospital CEO compensation. We found mixed evidence with respect to the significance of the association between financial performance and uncompensated care and CEO compensation. Among the other nonprofit performance measures, patient satisfaction was significantly associated with CEO compensation, but other measures were not significant determinants of CEO compensation. Our results suggest nonprofit hospitals balance their financial health against their mission when setting CEO incentives. Additional policy targeting transparency in hospital CEO compensation may be warranted to help policymakers understand the specific factors used by hospital boards to incentivize CEOs.

## Introduction

Privately owned nonprofit (henceforth nonprofit) hospitals, which comprise approximately 57% of community hospitals [1], are exempt from most federal, state, and local taxes in exchange for the provision of community benefit. Community benefit includes a range of activities such as provision of charity care, community health improvement services, and conducting research. Nonprofit hospitals spent approximately $62 billion on community benefit activities in 2011, which exceeded the estimated value of the nonprofit hospital tax exemption ($24.6 billion) [2,3]. However, community benefit spending represents a small share of total expenditures (9.7% in 2011), and has decreased following the passage of the Affordable Care Act (ACA) even as the ACA imposed additional community benefit reporting requirements on hospitals [2,4]. As a result, nonprofit hospitals face continued scrutiny over whether their community benefit activities adequately justify their tax-exempt status [5].

Empirical research has provided insight into the objectives valued by nonprofit hospitals through examining their community benefit spending and activities. Community benefit spending skews towards patient services such as charity care, subsidized health services, and unreimbursed costs, with less than 10% of community benefit spend going toward community health or contributions to community groups [6–8]. Furthermore, mean spending on community health initiatives does not vary across hospitals in states with different community benefit regulations [9]. While Singh et al. (2015) show community benefit spending on patient services predominates even among hospitals in counties with greater health needs, Franz et al. (2021) found that hospitals are more likely to invest in health needs highly ranked by their communities [10,11]. Several studies provide support that nonprofit hospitals adequately justify their tax exempt status because they provide more community benefit relative to for profit hospitals [12–16]. However, this result depends on how broadly researchers define community benefit, and many nonprofit hospitals do not provide incremental community benefit unless bad debt or unreimbursed costs are included in calculations [17–19].

This paper adds to the debate of what nonprofit hospitals value by examining the issue through another lens, in particular the compensation structure that the hospital board provides its chief executive officer (CEO). Nonprofit hospital CEO compensation has itself received considerable attention in light of nonprofits’ tax-favored status as well as the high costs of hospital care [20]. Several studies have considered the relationship between nonprofit hospital CEO pay and hospital performance [21], and our paper re-examines this issue using more recent data. To our knowledge we are the first to study this issue using post-ACA data, namely 2010 and 2015. Further, while the most recent studies of hospital CEO compensation relied on cross sectional data, our study strengthens the evidence base by exploiting a panel data set on compensation in 2010 and 2015 to address unmeasured time-invariant confounding. Since nonprofit hospitals may prioritize mission-related objectives (including community benefit provision) in addition to financial sustainability, we explore whether CEO pay is linked to hospital financial performance or nonprofit measures of performance, which include clinical quality, patient satisfaction, and the provision of uncompensated care. The results of our study can help policymakers understand what hospital boards seeks to incentivize from their leadership and whether this has shifted since the passage of the ACA.

## Background

### Nonprofit hospital CEO compensation

While no explicit limits on nonprofit CEO compensation exist, organizations must establish that compensation is reasonable through the rebuttable presumption process. Specifically, compensation must be approved by an independent governing body, supported by data from comparable organizations, and adequately documented [22]. In a 2006 study, the Internal Revenue Service (IRS) found a majority of respondent hospitals engaged in practices consistent with rebuttable presumption [23]. Although several states have considered placing limits on hospital CEO compensation, to date no legislation has been passed [24–26].

Nonprofit hospitals report CEO compensation as part of their annual filing requirements, yet compensation practices still lack transparency. Past surveys of hospitals conducted by the American College of Healthcare Executives (ACHE) indicate that hospital CEO compensation is based on a multitude of factors, including hospital financial performance, clinical quality, and patient satisfaction [27,28]. However, the language around these incentives–at least what is available in the public domain–is vague and lacks specific performance targets [29–31]. Moreover, empirical studies have not consistently found a strong link between nonprofit CEO compensation and many of the factors reported in ACHE surveys [21].

Across empirical studies, hospital size (usually measured by beds, although sometimes revenues are a proxy) is consistently positively associated with CEO compensation [21]. In contrast, evidence linking CEO compensation with hospital performance measures is mixed [21]. While hospital financial performance is generally positively associated with CEO compensation, one of the two most recent studies found no relationship between hospital financial performance and compensation [32,33]. Although some studies have included proxies for hospital quality such as nurses per patient day, Joynt et al. (2009) was the first to use 30-day readmissions and mortality as clinical quality measures [33]. However, they found no significant association between CEO compensation and quality, which is consistent with older studies that used other proxies for quality [21].

### Conceptual framework

A general literature on agency theory suggests that CEOs need to be incentivized to act on behalf of the board of directors (or, in the case of publicly traded companies, shareholders, who select the board) [34]. Compensation is therefore tied to organizational outcomes that are valued by the board and can be linked to CEO effort. While most companies are for-profit organizations, a substantial majority of hospitals are nonprofit [35]. This feature of the industry has invited the question of what nonprofit hospitals seek to maximize. The literature notes two polar cases: nonprofit hospitals altruistically serving their communities, versus nonprofit hospitals acting as “for-profits in disguise” [35,36]. In the latter case, we expect hospital financial performance will be the primary determinant of CEO compensation. In the former case, if nonprofit hospitals are purely altruistic, then only nonprofit performance measures–such as quality of care–should influence CEO compensation. In reality, nonprofit hospitals may represent a mixture of these polar cases. Empirical evidence on the behavior of nonprofit hospitals is mixed [36–40], but does not unambiguously support the “for-profits in disguise” view [41]. Moreover, while nonprofit hospitals tend to provide more unprofitable services and charity care than for-profits, they still balance financial objectives with social ones [42].

In the broader agency literature, empirical studies have shown CEO compensation is linked to firm financial performance, and this generally holds true in the hospital industry [21,43]. If, however, nonprofit hospitals value social welfare (e.g., quality) in addition to financial performance, we expect CEO compensation to be correlated with nonprofit performance measures such as 30-day mortality and readmissions rates. We also consider uncompensated care, a measure of community benefit, as another nonprofit performance measure. Unlike other nonprofit performance measures, the expected empirical relationship between uncompensated care and CEO compensation is ambiguous. Although uncompensated care may contribute to the hospital’s mission, it also creates financial losses. If hospital boards value financial performance more than achieving mission-driven outcomes, we may observe an insignificant or negative association between CEO compensation and uncompensated care. In contrast, if hospitals are willing to shoulder some degree of financial losses to support their mission-driven outcomes, CEO compensation and uncompensated care could be positively correlated.

## Data and methods

### Data

Our sample included US nonprofit, non-federal, short-term, general acute care hospitals with CEO compensation data for 2010 and 2015. We obtained compensation data for hospital CEOs from IRS Form 990 digitized by GuideStar [44]. We developed a crosswalk to link Employer Identification Number (EIN) with CMS certification number (CCN) to merge compensation data with our other data sources. We limited our analysis to CEOs who managed a single hospital and excluded system and regional CEOs since we expect facility and system CEOs to have different responsibilities and therefore different incentives and compensation packages. Our final CEO sample had compensation information for 1,605 CEOs across 1,485 hospitals in 2010 and 1,087 CEOs across 1,006 hospitals in 2015 (some hospitals in our data reported multiple facility-level CEOs; see Supplement S1 for details related to the crosswalk and data cleaning in S1 Appendix). Hospitals with compensation data have similar characteristics as all nonprofit general acute care hospitals in the American Hospital Association (AHA) Annual Survey Database (see S1 Table 1 in S1 Appendix, but are more likely to be system hospitals, more likely to be located in the Northeast, and less likely to be in the West (these differences are consistent with previous studies) [32,45].

The dependent variable was total CEO compensation, which equals the sum of three measures reported in Form 990: compensation from the primary organization (including salary and benefits), compensation from related organizations (e.g., foundation associated with the hospital), and other compensation.

Our independent variables of interest reflect potentially important determinants of CEO compensation. Following the literature [21,43], total margin (the ratio of net income to total revenue) was our primary measure of hospital financial performance, and was obtained from the Healthcare Cost Report Information System (HCRIS) [46]. In sensitivity analysis, we considered retained earnings, measured as total assets minus total liabilities, divided by total expenditures, again from HCRIS. We also considered three measures of nonprofit performance: clinical quality, patient satisfaction, and uncompensated care. Clinical quality measures included hospital mortality and readmission rates developed and reported by CMS. Specifically, we applied CMS’s risk-adjustment algorithms for acute myocardial infarction (AMI), congestive heart failure (CHF), and pneumonia to a random 20% sample of Medicare fee-for-service claims [47]. We then created hospital-level composite mortality and readmissions measures by calculating the weighted average across the three conditions [48]. Composite measures made our models more parsimonious, but models with individual quality measures generated similar conclusions (see S1 Table 6 in S1 Appendix). Patient satisfaction was measured by the percentage of patients that would recommend a given hospital from Hospital Consumer Assessment of Healthcare Providers and Systems (HCAHPS) surveys [49]. Uncompensated care, or the total monetary value of care for which no payment was received, was obtained from HCRIS and divided by the total monetary value of care provided (paid and uncompensated).

Hospital characteristics were taken from the AHA Annual Survey Database. Number of beds was included as a proxy for organization size, which has been shown to be an important determinant for CEO compensation [21]. We also included the number of full time equivalent (FTE) employees, which also proxies for size, but may also capture organizational complexity. Finally, we included the shares of discharges attributable to Medicare and Medicaid patients, as well as indicator variables for teaching status, system membership, rurality, and whether the hospital is located in a Medicaid expansion state. All financial variables were inflated to 2015 dollars using the consumer price index. All independent variables were measured in the year prior to CEO compensation. Table 1 presents summary statistics. As shown in the appendix (S1 Tables 4–5 in S1 Appendix), the non-financial performance measures (mortality, readmissions, willingness to recommend and uncompensated care) do not show evidence of multicollinearity.

**Table 1:** Summary statistics.

Variable name	Description	Mean (St. Dev)	Min	Max
CEO Compensation	Total hospital CEO compensation, 2010 & 2015 in 2015 USD (millions)	1.126 (0.896)	0.05	6.52
Total Margin	Ratio of net income to total revenue	4.85 (6.12)	-41.2	37.0
Mortality	Risk-adjusted percentage of qualifying admissions for AMI/CHF/pneumonia resulting in death within 30 days of hospital admission	12.60 (0.798)	10.4	15.9
30-Day Readmissions	Risk-adjusted percentage of qualifying admissions for AMI/CHF/pneumonia resulting in unplanned readmission to any acute care hospital within 30 days of discharge from hospitalization	19.97 (0.918)	17.6	22.7
Recommend	Risk-adjusted percentage of patients who would recommend a hospital to friends and family (9–10 response)	71.23 (8.85)	28.0	91.0
Uncompensated Care	Total monetary value of care for which no payment was received (share of total value of care)	0.080 (0.053)	0.005	0.450
Beds	Total number of beds	489.5 (346.0)	9.0	1,576
FTE Employees	Total number of full time equivalent employees	3,410 (3,564)	100	25,856
System Hospital	Indicator equal to 1 if hospital is part of a hospital system	0.510 (0.500)	0	1
Teaching Hospital	Indicator equal to 1 if hospital is a teaching hospital	0.290 (0.454)	0	1
Rural Hospital	Indicator equal to 1 if hospital is in a rural location	0.130 (0.336)	0	1
% Medicare Discharges	Hospital discharges accounted for by Medicare patients (%)	0.448 (0.099)	0	1
% Medicaid Discharges	Hospital discharges accounted for by Medicaid patients (%)	0.203 (0.100)	0	1
Northeast Region	Indicator equal to 1 if hospital is located in the Northeast Census region	0.296 (0.456)	0	1
South Region	Indicator equal to 1 if hospital is located in the South Census region	0.400 (0.490)	0	1
Midwest Region	Indicator equal to 1 if hospital is located in the Midwest Census region	0.219 (0.414)	0	1
West Region	Indicator equal to 1 if hospital is located in the West Census region	0.085 (0.279)	0	1
Medicaid expansion state	Indicator equal to 1 if hospital is located in a Medicaid expansion state as of 2015 [50]	0.247 (0.431)	0	1

Notes: CEO compensation data are for 2010 and 2015 pooled. All variables other than compensation are measured in the year prior to compensation since we used lagged hospital performance measures in our regressions (see Eq 1 in Analysis section). Summary statistics are weighted by beds. Mortality and 30-day readmissions are composite measures that are equal to the weighted average of the respective measures for AMI, CHF, and pneumonia. S1 Table 2 and S1 Table 3 in S1 Appendix compare these summary statistics across different analysis samples.

### Analysis

We used the following econometric framework to determine the relationship between CEO compensation and hospital performance measures:

$${\text{ln}\left(CEO\;comp\right)}_{iht}=\beta_{0}+\beta_{1}{\left(financial\;performance\right)}_{h\left(t-1\right)}+{\left(nonprofit\;performance\right)}_{h\left(t-1\right)}\gamma +Z_{h(t-1)}\delta +\theta_{t}+\theta_{h}+\theta_{i}+\epsilon_{iht},$$ (1)

where *i* indexes CEO, *h* indexes hospital, and *t* indexes year; nonprofit performance is a vector that includes clinical quality, patient satisfaction, and uncompensated care, Z_h(t-1)_ are hospital characteristics, θ_t_ is a year (2015) indicator, θ_h_ are hospital indicator variables, and θ_i_ are CEO indicator variables used in some analyses. Following the existing executive compensation and firm performance literature, we use lagged (i.e., corresponding to 2009 and 2014) rather than contemporaneous hospital performance measures to help mitigate the question of whether higher compensation leads to improved performance or improved performance results in higher compensation [43,51].

Even though we control for hospital characteristics and state, there may be concern that our estimates are biased due to omitted hospital-specific factors (such as patient population or economic shocks). Ideally, we would leverage a natural experiment or some other exogenous change in hospital performance to identify the causal effect of hospital performance on CEO compensation. However, absence of natural experiments reflects a shortcoming of the hospital CEO compensation literature generally. We attempt to address this issue by exploiting the panel nature of the data and estimate hospital as well as CEO fixed effects models to control for unobserved time-invariant characteristics that drive both compensation and hospital performance simultaneously.

## Results

**Fig 1:**
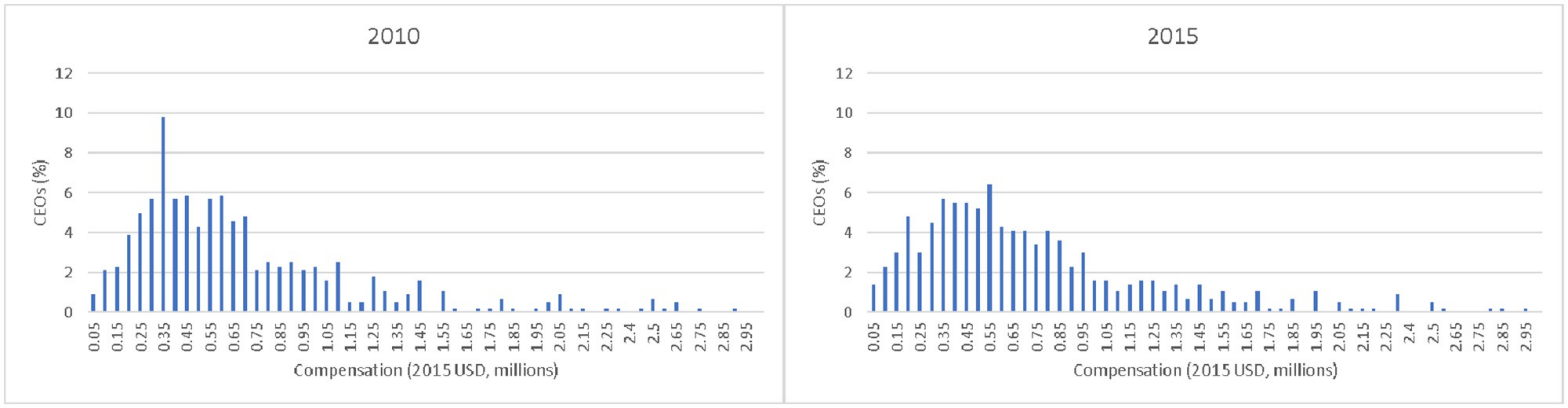
CEO compensation by year.

2,431 hospitals (1,446 and 985 in 2010 and 2015, respectively) had non-missing data for CEO compensation, total margin, and hospital characteristics. Prior to dropping hospitals with missing nonprofit performance data, mean (unweighted) hospital CEO compensation for 2010 and 2015 pooled was $630,000. After dropping hospitals with missing nonprofit performance data, mean hospital CEO compensation was $746,371. The pattern of higher CEO compensation for hospitals with non-missing data reflects the fact that hospitals with missing nonprofit performance were more likely to be smaller, more likely to be in rural areas, and less likely to be teaching hospitals. Histograms for hospital CEO compensation in 2010 and 2015 for the balanced panel used in our fixed effects estimation are shown in Fig 1; mean (median) compensation was $788,469 ($590,548) in 2010 and $809,143 ($629,358) 2015, measured in 2015 dollars. We provide a comparison of hospital characteristics and compensation summary statistics for all analysis samples in the appendix (S1 Tables 2–3 in S1 Appendix).

The effects of hospital performance measures on CEO compensation are presented in Table 2. Columns 1 and 2 show the results for specifications that include hospital financial performance (total margin) and nonfinancial performance measures separately, both with hospital fixed effects. Column 3 presents our primary specification, which corresponds to Eq (1).

**Table 2:** Effect of hospital performance measures on CEO compensation (2010 & 2015 fixed effects regression results).

	(1) Margin only	(2) Quality metrics only	(3) Margin + quality, hospital fixed effects	(4) Margin + quality, CEO fixed effects
Total Margin	0.0063* (0.0038)		0.0061 (0.0039)	0.0074 (0.0066)
Retained Earnings				
Mortality		-0.0572 (0.0397)	-0.0568 (0.0393)	-0.0286 (0.0382)
30-day Readmissions		0.0956 (0.0789)	0.0928 (0.0787)	-0.1075 (0.0911)
Recommend		0.0098* (0.0052)	0.0097* (0.0052)	-0.0014 (0.0049)
Uncompensated Care		0.3458 (0.4677)	0.2934 (0.4662)	-0.0605 (0.4554)
N	882	882	882	330
Same CEO both years?	No	No	No	Yes
Estimation	FE (Hospital)	FE (Hospital)	FE (Hospital)	FE (CEO)

Notes: Dependent variable is natural log of CEO compensation. Standard errors clustered at the hospital level in parenthesis. All regressions include the hospital characteristics listed in Table 1 and a year indicator. S1 Table 6 in S1 Appendix provides estimated coefficients and standard errors for the hospital characteristics.

Significance:

***p<0.01;

**p<0.05;

*p<0.1.

For the specification without nonprofit performance measures, mean CEO compensation increases by 0.638% (p = 0.093; which corresponds to roughly $3,969) for a 1 percentage-point change in total margin. In the specification with both total margin and nonprofit performance measures (column 3), the effect of a 1 percentage-point increase in total margin is similar, 0.610, but no longer statistically significant (p = 0.12). Our results are similar if we use retained earnings as an alternative measure for financial performance: mean compensation rises 0.196% with a 1 percentage-point increase in retained earnings (p = 0.009). While retained earnings is statistically significant the standardized effect size is small relative to that of total margin: a one-standard deviation increase in retained earnings increases CEO pay by 0.11% compared with 4% for a one-standard deviation increase in total margins (4%). Specification (4) limits the sample to the subset of hospitals with the same CEO in 2010 and 2015, and includes CEO fixed effects. Under this model, financial performance (again based on the primary measure of total margin) is not significant.

Among the nonprofit performance measures, only the recommend variable is significant in any of the specifications with both financial and nonprofit performance (specifically, specifications 3 and 4, though not the CEO fixed effect specification.) Since nonprofit hospitals receive tax exempt status in part for their community benefit provision, we might expect CEO compensation to be positively correlated with uncompensated care, yet we find it is not a statistically significant determinant of compensation. Alternatively, it is possible that CEO compensation is nonlinear in uncompensated care: for example, compensation may increase with uncompensated care up to some level, then decreases as the financial downside outweighs the benefits from its provision. We estimated model specifications that included quadratic terms for all performance measures, but no quadratic terms were statistically significant.

Since the passage of the ACA, more emphasis has been placed on quality, but hospitals have also faced increasing pressure to control costs. However, we did not find any evidence of differential effects by year (Table 3). The relative stability of our results across years likely reflects the fact that CEO employment contracts tend to have durations longer than three years with a majority containing automatic extension clauses [28].

**Table 3:** Effect of hospital performance measures on CEO compensation by year.

	2010	2015	t-stat
Total Margin	0.0005 (0.0047)	0.0089** (0.0045)	1.52
Mortality	-0.0728 (0.0465)	-0.0378 (0.0432)	0.88
30-day Readmissions	0.0892 (0.0827)	0.1022 (0.0813)	0.23
Recommend	0.0095* (0.0051)	0.0121** (0.0061)	0.68
Uncompensated Care	0.0156** (0.5619)	0.6974 (0.7819)	0.67

Notes: Marginal effects from a hospital fixed effects regression specification that includes the same variables as specification (3) from Table 2 plus interaction terms between the hospital performance measures and year. Standard errors clustered at the hospital level in parenthesis; t-statistic is for the null hypothesis that the marginal effect in 2010 and 2015 is the same.

Significance:

***p<0.01;

**p<0.05;

*p<0.1.

### Sensitivity checks

Results for three alternative estimation strategies are presented in Table 4 (the first two specifications provide the results that correspond to columns 3 and 5 in Table 2 for reference). To begin with, we estimate pooled OLS models with state and hospital referral region (HRR) indicators for geographic controls for hospitals with data for both 2010 and 2015. In both cases, higher margins are associated with significantly higher compensation. With state fixed effects (specification 3), compensation is positively associated with willingness to recommend (p = 0.030), but *negatively* associated with uncompensated care (p = 0.041). These results would be confounded if within-area (that is, hospital- or CEO-level) changes in unmeasured determinants of compensation were correlated with changes in hospital performance. These specifications do provide a more direct comparison with previously published studies.

**Table 4:** Alternative estimation results: Effect of hospital performance measures on CEO compensation.

	(1) Main specification (Hospital level)	(2) Main specification (CEO level)	(3)	(4)	(5)
Total Margin	0.0061 (0.0039)	0.0074 (0.0066)	0.0072* (0.0039)	0.0082* (0.0049)	0.0038 (0.0064)
Mortality	-0.0568 (0.0393)	-0.0286 (0.0382)	0.0148 (0.0284)	0.0479 (0.0351)	0.0204 (0.0459)
30-day Readmissions	0.0928 (0.0787)	-0.1075 (0.0911)	0.0267 (0.0393)	-0.0094 (0.0425)	0.1860** (0.0908)
Recommend	0.0097* (0.0052)	-0.0014 (0.0049)	0.0069** (0.0032)	0.0045 (0.0036)	0.0030 (0.0066)
Uncompensated Care	0.2934 (0.4662)	-0.0605 (0.4554)	-1.218** (0.5930)	-0.8180 (0.6010)	-1.286* (0.7050)
Geographic controls			State	HRR	HRR
Estimation	FE (Hospital)	FE (CEO)	OLS	OLS	OLS
Same CEO both years?	No	Yes	No	No	Yes
N	882	330	882	882	330

Notes: Dependent variable is natural log of CEO compensation. Standard errors clustered at hospital level in parenthesis. All regressions include the hospital characteristics listed in Table 1, a year indicator, and indicators for the geographic controls noted in the table. Columns 1–2 present the main specification results (columns 3–4 in Table 2) for reference. The results in columns 3–4 do not change much if we include an indicator for CEO turnover and are available upon request.

Next, we estimate the pooled OLS model with HRR indicators and limit the sample to hospitals with the same CEO in 2010 and 2015. The coefficient on total margin remains positive but is no longer significant, as it was in specifications 3 and 4 (state and HRR fixed effects on full sample.) Willingness to recommend is insignificant, in contrast with the positive coefficients for specifications 1 and 3 (hospital and state fixed effects, respectively). Compared to specification 3, compensation continues to be negatively associated with uncompensated care (p = 0.070); however, this result should be interpreted with caution in light of the relatively large standard errors. In contrast with the other specifications in the table, compensation is positively associated with readmissions (p = 0.042).

Our results are similar based on other sensitivity checks reported in the Appendix (S1 Tables 7–9 in S1 Appendix), including how we measured independent variables and the inclusion of additional alternative measures of financial performance.

## Discussion

We examined potential determinants of nonprofit hospital CEO compensation in 2010 and 2015. We found that financial performance has a consistently positive association with CEO compensation, although this relationship was insignificant in some specifications. With respect to nonprofit performance, neither mortality nor readmissions were associated with compensation in analyses that addressed unmeasured time-invariant confounding. Willingness to recommend was positively associated with compensation in some specifications. Notably, our most reliable analyses did not indicate that CEO compensation increases with the provision of uncompensated care. Indeed, in some sensitivity analyses, there was a significant *negative* relationship.

Our results are generally consistent with previous hospital CEO compensation studies. Most recently, Joynt et al. used data from 2009 and found no relationship between total margin or nonprofit performance measures and CEO compensation [33]. Since more emphasis has been placed on improved quality since the passage of the ACA [52–54], we might expect to find empirical evidence that quality is significant determinant for CEO pay in our study, which included data from 2015. However, we find that even with more recent data, hospital mortality and readmissions remain an empirically insignificant determinant for CEO compensation in our most reliable analyses (willingness to recommend is positively associated, depending on the specification). The lack of empirical evidence of a robust and comprehensive link between CEO compensation and nonprofit performance might be explained in a few ways. First, it is possible that hospital CEO compensation is tied to hospital quality outcomes other than those reported to CMS and used in our study. Second, it is possible that hospital boards recognize that quality outcomes are more correlated with patient characteristics or factors other than CEO performance and therefore do not closely tie CEO compensation to these measures [55,56].

Importantly, although provision of charity care (which includes uncompensated care) represents a key activity for nonprofit hospitals to maintain their tax-exempt status, we found that CEO compensation is not positively associated with uncompensated care. While nonprofit hospitals must balance mission-based objectives, including the provision of charity care, against financial viability, our results suggest hospital boards prefer CEOs to focus on financial performance relative to uncompensated care. Several studies support this hypothesis for at least a subset of nonprofits: seven of the ten most profitable hospitals in 2013 were nonprofit, and charity care provision was disproportionately low among hospitals in the top income quartile [57,58].

Although it is perhaps surprising to see weak or inconsistent empirical relationships between key hospital performance measures and CEO compensation, it is not obvious that this should cause concern. While some might argue pay-for-performance could lead to improved hospital (and patient) outcomes, research suggests pay-for-performance for CEOs might not work well in practice [59]. In particular, pay-for-performance may work best for routine tasks, which seemingly would not apply to the role of a CEO. Moreover, CEOs that have compensation tied closely to certain performance metrics might be more likely to behave unethically to meet their goals. As a result, pay-for-performance could induce safety issues or financial misreporting that would not occur otherwise. Thus, while regulations that aim to link CEO pay to specific outcomes (such as community benefit) may seem attractive, they are likely to come with unintended consequences spurred by CEOs or hospital boards attempting to circumvent regulations [60].

A recent study found that nonprofit hospital CEOs earn substantially less than CEOs of publicly traded non-hospital healthcare corporations, such as health insurers or pharmaceutical manufacturers [45]. Although this finding is consistent with the theory that nonprofit employees earn less than their for-profit counterparts, legislation that aims to regulate hospital CEO pay through caps may harm hospitals if it results in talented CEOs leaving for positions in other firms. Our study suggests that before policymakers can determine whether hospital CEOs earn “too much,” we need to better understand their compensation packages and how they are determined. For example, in their 10-K filings, publicly traded companies are required to report each element of CEO compensation packages (e.g., base salary, bonuses, incentive compensation, etc.) and provide a justification for why each element is included and what it is designed to reward [61]. In contrast, nonprofit hospitals might not always report detailed elements of compensation, and in some cases may hide compensation through legal exemptions [31,62,63].

While surveys conducted by ACHE indicate many elements, including quality, are important for hospital CEO pay and evaluation, our results and past studies do not support their claims. Of note, only 18% of hospitals reported using activities to improve community or population health as a factor in incentive pay [28], which generally aligns with our finding for uncompensated care. With respect to quality measures, lack of significance is potentially a data limitation because we only observe quality measures reported to CMS. It is possible that hospitals have their own internal quality measures used for CEO compensation. If this is the case, public reporting of such measures in Form 990 would improve our understanding of how compensation packages are derived. Moreover, if most hospitals are using quality measures that are different from publicly reported measures, CMS might use that information to improve public reporting requirements or reimbursement decisions.

Our study has several limitations. Although economic theory indicates CEO characteristics such as education are important factors for compensation, we do not observe CEO characteristics in the data. We attempt to address this through a sensitivity analysis which limits the sample to hospitals with the same CEO in both years and includes CEO fixed effects. While the CEO fixed effects analysis focuses on hospitals with the same CEOs in 2010 and 2015, we cannot be certain that the CEOs were the same in 2009 and 2014, when hospital performance was measured. If the annual rate of CEO “attrition” were constant between 2010 and 2015, then 78% of CEOs in 2009 would have remained CEO in 2010. A second limitation is that we do not observe compensation components (e.g., base salary, bonus, etc.). It is possible, for example, that certain hospital performance measures are more closely tied to bonuses than base salary. Consequently, if base salary represents a substantial proportion of total compensation, we are less likely to find a strong relationship between those performance measures and total compensation. While understanding the impact of hospital performance on components of CEO pay is interesting, this cannot be addressed until better data become publicly available. Third, clinical quality measures are specific to three conditions in the Medicare population and might not generalize to other conditions or the non-Medicare population. However, these conditions are important for care delivery, and were the earliest conditions selected by CMS for public reporting and reimbursement.

## Conclusion

Hospital CEO compensation has been scrutinized since a 2006 IRS study [23], and while reporting practices have improved, hospital CEO compensation still lacks transparency. Our study confirms that the compensation of CEOs in nonprofit hospitals has a stronger relationship with the financial performance of these hospitals than with nonprofit performance measures. While this result is perhaps counter to the idea that nonprofit hospitals value mission-related outcomes such as community benefit, it does reflect hospitals balancing their financial health against their mission. Additional policy targeting transparency in hospital CEO compensation may be warranted. More transparency and better data on compensation packages, including how compensation is tied to specific hospital performance measures, could help policymakers understand the specific factors used by hospital boards to incentivize CEOs and by extension what objectives they value.

## Supporting information

S1 Appendix (DOCX)

S1 Dataset (CSV)
