# Supplementary material for: What do nonprofit hospitals reward? An examination of CEO compensation in nonprofit hospitals
Source: PLoS One. 2022 Mar 21;17(3):e0264712. doi: 10.1371/journal.pone.0264712 (PMC8936479; doi:10.1371/journal.pone.0264712)
Supplement: S1 Appendix — (DOCX) [file pone.0264712.s001.docx]

**What do Nonprofit Hospitals Reward? An Examination of CEO Compensation in Nonprofit Hospitals**

**Appendix**

**Sample Construction**

We created our hospital-level CEO compensation dataset from GuideStar data with National Taxonomy of Exempt Entities (NTEE) codes equal to E20-E22. These data were matched to hospital characteristics from the AHA Survey Database (henceforth AHA data), financial information from HCRIS, and quality variables from Hospital Compare. The initial GuideStar data corresponding to the relevant NTEE codes (2010 and 2015 pooled) included information on 66,107 employees across 7,687 hospitals. Our final sample of CEOs were identified using keywords in the employee title field, and we limited the sample to employees at organizations that could be matched to observations in the AHA Survey Database using a crosswalk that mapped employer identification number (EIN) to CMS certification number (CCN).

**Hospital Crosswalk Overview**

We created a crosswalk based on methodology described on the Center to Advance Community Health & Equity (CACHE) website to map EIN to CCN, which allowed us to link GuideStar data (which uses EIN to identify hospitals) with AHA data.[1] We first matched based on organizations’ latitude and longitude within two decimal places.^[[1]](#footnote-1)^ Hospital addresses were extracted from GuideStar and standardized using Google’s geocoding application programming interface (API); latitude and longitude are reported in the AHA data. Second, we matched hospitals based on exact city and state and similar hospital name, second name, or DBA matches.^[[2]](#footnote-2)^ Similar names were defined as those having a similarity score >85%, and matches with similarity score between 70% and 85% were checked manually.^[[3]](#footnote-3)^ We chose this matching algorithm because it was better able to match strings with similar keywords but different lengths, whereas a distance formula would not have performed as well.

Potential matches were subjected to several checks. Manual confirmation of matches was completed using a Google search of the GuideStar hospital name and address, which allowed us to verify whether the organization was a hospital. First, we manually checked 741 cases where the matching methods described yielded multiple initial matches; we dropped or corrected 477 of these cases, and retained all valid matches. Second, we checked potential latitude/longitude matches that were farther than 0.25 miles apart (110 cases) and ultimately dropped 59 of these matches. Finally, we checked potential matches that had dissimilar names or different cities or states (643 cases). Valid explanations for such cases include hospitals that report under multiple names or file in different cities from where they operate; 277 of these cases were dropped. Additionally, we checked 292 cases where a potential match combination varied across 2010 and 2015; of these 264 were dropped or updated. In the process of verifying matches, 338 non-hospitals were identified in the GuideStar data and dropped from our sample. After completing all checks, 3,694 CCNs were identified for 3,742 EINs (match rate equal to 98.7%).

**Exclusion Criteria**

We excluded employee observations if the employee worked for an organization that was not based in the US or if the employee was missing compensation information. Second, we excluded observations that are foundations and could not be matched to a hospital in the AHA data based on our crosswalk. Foundations were identified using keywords (“foundation,” “fndn,” or “fdn”) in the organization name field. Finally, some organizations in Guidestar are categorized as hospitals according to NTEE code, but are not hospitals. Therefore, we excluded any observations for hospital organizations that do not deliver short-term acute care or have a missing value for CCN.

**CEO Sample Identification**

We flagged observations as CEOs using an initial set of keywords and their variants (e.g., “ceo” or “chief executive”) which were applied to the employee title field. We applied another set of keywords to reclassify individuals but were flagged based on initial keywords (e.g., “secretary to ceo”). We also applied another set of keywords to capture possible CEOs from organizations that had none flagged based on the initial keywords (e.g., “exec director”). After applying keywords, 81% of hospital-year observations had exactly one CEO flagged; 9% had no CEO flagged, and 10% had two more CEOs flagged.

We then manually checked the personnel data for hospital-year observations with either zero or two or more flagged CEOs to identify the hospital-specific CEO for those observations. For hospitals without a CEO, if exactly one employee with the title of chief administrative officer, administrator, or director was reported, we flagged that employee as the CEO. 7% of hospitals did not report any CEO, and these were excluded from the sample.

In cases where a hospital-year had multiple CEOs flagged, we excluded system and regional-level CEOs using title keywords and by comparing titles across CEOs within a hospital-year to determine which CEO was the facility CEO. In some cases, a hospital-year had more than one facility-level CEO; this occurred for one of two reasons: 1) different CEOs served during a subset of the year (“partial year CEO”) or 2) the hospital reported two individuals with identical titles (“multiple CEOs”). Hospital-year observations were flagged as having a partial CEO if one or more of the employees’ title contained dates of employment or keywords “resigned,” “part yr,” “terminated,” or “retired,” “eff. from,” “beg.,” “through,” or “end.”

Rather than keep two or more CEOs per organization in our sample for some hospitals, we combined their compensation data into a single observation. Specifically, we summed compensation across partial CEOs within a hospital-year and took the maximum compensation for multiple CEOs. After applying keywords and manually checking and reclassifying observations, we had compensation information for 1,605 CEOs across 1,485 hospitals in 2010 and 1,087 CEOs across 1,006 hospitals in 2015.

**Comparison of Guidestar CEO Sample to AHA Nonprofits**

Hospitals with facility-level CEO compensation data account for approximately 46% of all non-profit hospitals in the AHA in 2010 and 2015. Hospitals with CEO compensation data have similar characteristics as all non-profit hospitals in the AHA (Table 1), but are less likely to be system hospitals, more likely to be located in the Northeast census region and less likely to be located in the West census region. These differences are consistent with previous studies.[2,3]

Table 1. Mean Hospital Characteristics for Nonprofit Hospitals in AHA Survey versus CEO Sample

|  | AHA – Nonprofit [N=5,423] | CEO Sample [N=2,491] | AHA Out of Sample [N=2,932] |
| --- | --- | --- | --- |
| Beds | 191 | 203* | 181 |
| FTE employees | 1,199 | 1,295* | 1,117 |
| System hospital | 0.66 | 0.46* | 0.84 |
| Teaching hospital | 0.07 | 0.08* | 0.06 |
| Rural hospital | 0.38 | 0.42* | 0.35 |
| % Medicaid discharges | 0.18 | 0.18* | 0.17 |
| % Medicare discharges | 0.51 | 0.51* | 0.51 |
| Northeast region | 0.18 | 0.23* | 0.14 |
| South region | 0.29 | 0.28 | 0.29 |
| Midwest region | 0.35 | 0.35 | 0.35 |
| West region | 0.18 | 0.13* | 0.22 |

Notes: Data for 2010 and 2015 are pooled. CEO sample consists of hospitals with non-missing hospital CEO compensation data in Guidestar.

*t-test for difference in means for CEO sample and AHA out of sample are significant at p<0.1.

**Sample Comparisons**

We dropped hospitals that did not have data for at least one hospital performance measure. Less than 3% of the CEO sample has missing values for total margin or hospital characteristics. Substantially more hospitals are missing data for at least one nonprofit performance measure – incorporating these measures into our analysis reduces our sample by approximately 30%. A comparison of hospital characteristics and performance measures across samples is provided in Table 2.

Table 2. Mean Hospital Characteristics and Performance Measures

|  | CEO Sample [N=2,491] | Sample 1  [N=2,431] | Sample 2  [N=1,664] | Balanced panel [N=882] | Same CEO  [N=330] |
| --- | --- | --- | --- | --- | --- |
| Beds | 203 | 204 | 253* | 277^x^ | 261^y^ |
| FTE employees | 1,295 | 1,277 | 1,615* | 1,821^x^ | 1,628^y^ |
| System hospital | 0.463 | 0.432 | 0.463* | 0.397^x^ | 0.330^y^ |
| Teaching hospital | 0.084 | 0.079 | 0.099* | 0.112^x^ | 0.097 |
| Rural hospital | 0.420 | 0.418 | 0.310* | 0.285^x^ | 0.282 |
| % Medicare discharges | 0.513 | 0.504 | 0.486* | 0.479^x^ | 0.494^y^ |
| % Medicaid discharges | 0.173 | 0.174 | 0.187* | 0.191^x^ | 0.187 |
| *Hospital Performance Measures* | | | | | |
| Total margin |  | 3.32 | 3.86* | 4.10 | 4.37 |
| Mortality |  |  | 12.8 | 12.7^x^ | 12,7 |
| Readmissions |  |  | 20.0 | 19.9 | 20.0^y^ |
| Recommend |  |  | 70.0 | 70.0 | 70.4 |
| Uncompensated care |  |  | 0.079 | 0.078 | 0.078 |

Notes: Hospitals with missing total margin or hospital characteristics are dropped from the CEO sample to obtain Sample 1. Sample 2 additionally drops hospitals with missing data for any quality measure (mortality, readmissions, recommend, uncompensated care).

*t-test for difference in means for Sample 1 and Sample 2 is significant (p<0.1).

^x^t-test for difference in means for Sample 2 and balanced panel is significant (p<0.1).

^y^ t-test for difference in means for balanced panel and same CEO samples is significant (p<0.1).

Hospitals that are missing quality data are somewhat smaller (both in terms of beds and FTE employees) and less likely to be located in rural areas. Mean financial measures are similar across Samples 1 and 2. The loss of smaller and more rural hospitals in samples that have non-missing quality results in a different distribution of hospital CEO compensation compared with the sample of hospitals that have non-missing total margin and hospital characteristics (Sample 1).

Across all samples, the distribution of hospital CEO compensation does not change much between 2010 and 2015. We compare summary statistics for compensation across samples in Table 3.

Table 3. CEO Compensation Summary Statistics

|  | N | Mean | Std Dev | 25th percentile | 50th percentile | 75th percentile |
| --- | --- | --- | --- | --- | --- | --- |
| CEO Sample | 2,491 | 628,573 | 640,519 | 245,483 | 432,937 | 770,734 |
| Sample 1 | 2,431 | 630,000 | 637,986 | 247,331 | 435,978 | 774,655 |
| Sample 2 | 1,664 | 746,371 | 679,495 | 349,006 | 556,871 | 898,240 |
| Balanced Panel | 882 | 798,806 | 677,809 | 386,524 | 607,655 | 977,180 |
| Same CEO | 330 | 826,130 | 620,857 | 436,061 | 658,101 | 1,033,812 |

Notes: All values are in 2015 USD. Hospitals with missing total margin or hospital characteristics are dropped from the CEO sample to obtain Sample 1. Sample 2 additionally drops hospitals with missing data for any quality measure (mortality, readmissions, recommend, uncompensated care).

**Independent Variables**

For our main analysis, we created composite measures for mortality and 30-day readmissions by taking the weighted average across the three conditions (acute myocardial infarction (AMI), congestive heart failure (CHF), and pneumonia.) We present correlations for these variables in Table 4; correlations for all hospital performance measures, including the composite mortality and readmissions measures are given in Table 5.

Table 4. Correlations for Quality Components

|  |  | Mortality | | | Readmissions | | |
| --- | --- | --- | --- | --- | --- | --- | --- |
|  |  | AMI | CHF | Pneum | AMI | CHF | Pneum |
| Mortality | AMI | 1.000 |  |  |  |  |  |
|  | CHF | -0.203 | 1.000 |  |  |  |  |
|  | Pneum | 0.087 | 0.214 | 1.000 |  |  |  |
| Readmissions | AMI | 0.702 | -0.394 | 0.015 | 1.000 |  |  |
|  | CHF | -0.012 | -0.061 | -0.004 | 0.054 | 1.000 |  |
|  | Pneum | 0.490 | -0.313 | 0.056 | 0.678 | 0.129 | 1.000 |

Notes: Correlations are for the main estimation sample (balanced panel; N=882) AMI=acute myocardial infarction; CHF=congestive heart failure; Pneum=pneumonia

Table 5. Correlations for Independent Variables

|  | Total margin | Mortality | Readmissions | Recommend | Uncompensated care |
| --- | --- | --- | --- | --- | --- |
| Total margin | 1.000 |  |  |  |  |
| Mortality | -0.007 | 1.000 |  |  |  |
| Readmissions | -0.143 | 0.007 | 1.000 |  |  |
| Recommend | 0.227 | -0.065 | -0.166 | 1.000 |  |
| Uncompensated care | -0.188 | 0.031 | 0.279 | -0.371 | 1.000 |

Notes: Correlations are for the main estimation sample (balanced panel; N=882)

**Additional Results**

Table 6. Regression Coefficients for Hospital Characteristics

|  | (1) | (2) | (3)  Main specification (Hospital level) | (4)  Main specification (CEO level) |
| --- | --- | --- | --- | --- |
| Beds | -0.0004  (0.0006) | -0.0005  (0.0006) | -0.0005  (0.0005) | -0.0005  (0.0000) |
| FTE Employees | -0.00005  (0.00006) | -0.00004  (0.00006) | -0.00005  (0.00006) | -0.00002  (0.00008) |
| System Hospital | -0.1140  (0.0905) | -0.1020  (0.0887) | -0.0980  (0.0890) | -0.0164  (0.122) |
| Teaching Hospital | 0.5410*  (0.3010) | 0.5910**  (0.3010) | 0.5850**  (0.2970) |  |
| Rural Hospital | -0.0671  (0.1420) | -0.0813  (0.1290) | -0.0817  (0.1270) | -0.1020  (0.1350) |
| % Medicare Discharges | 0.0196  (0.2700) | -0.0291  (0.2780) | -0.0014  (0.2750) | -0.3060  (0.2650) |
| % Medicaid Discharges | -0.4140  (0.4610) | -0.3370  (0.4650) | -0.3320  (0.4610) | -0.3600  (0.3460) |
| Medicaid Expansion State | 0.1090*  (0.0560) | 0.1090*  (0.0570) | 0.1140**  (0.0567) | 0.0840  (0.0588) |
| Included hospital performance measures | Total margin | Nonprofit performance | Total margin,  Nonprofit performance | Total margin,  Nonprofit performance |
| N | 882 | 882 | 882 | 330 |
| Same CEO both years? | No | No | No | Yes |
| Estimation | FE (Hospital) | FE (Hospital) | FE (Hospital) | FE (CEO) |

Notes: Estimated coefficients for hospital characteristics correspond to regression results presented in Table 2 of the main paper. Dependent variable is natural log of CEO compensation. Standard errors clustered at the hospital level in parenthesis. All regressions include a year indicator. Teaching status is time invariant for the same CEO sample and excluded from the model. These results do not change much if we include an indicator variable for CEO turnover, and are available upon request. Significance: ***p<0.01; **p<0.05; *p<0.1

**Sensitivity Analyses**

Since we found no significant effect of the composite clinical quality measures on CEO compensation, we estimated regressions with the underlying components for quality and productivity rather than composite variables to check whether any individual components were statistically significant.

Table 7. Sensitivity Analysis: Regressions with Clinical Quality Components

|  | (1) | (2) | (3) |
| --- | --- | --- | --- |
| Total margin | 0.0063  (0.0039) | 0.0061  (0.0039) | 0.0062  (0.0039) |
| AMI mortality | -0.0274  (0.0270) |  | -0.0281  (0.0270) |
| CHF mortality | -0.0141  (0.0241) |  | -0.0152  (0.0242) |
| Pneumonia mortality | -0.0198  (0.0184) | 0.0312  (0.0301) | -0.0226  (0.0188) |
| AMI readmissions |  | 0.0091  (0.0518) | 0.0157  (0.0513) |
| Pneumonia readmissions |  | 0.0312  (0.0301) | 0.0362  (0.0308) |
| Patient satisfaction | 0.0099*  (0.0052) | 0.0091*  (0.0051) | 0.0099*  (0.0052) |
| Uncompensated care | 0.3300  (0.4720) | 0.2660  (0.4670) | 0.3020  (0.4690) |
| N | 882 | 882 | 882 |

Notes: Models are estimated using hospital fixed effects. Standard errors clustered at the hospital level in parenthesis. All regressions include hospital characteristics and an indicator for year. Coefficient on CHF readmissions could not be estimated due to multicollinearity. Significance: ***p<0.01; **p<0.05; *p<0.1

We also considered rating – the proportion of patients who rated the hospital a 9 or 10 (on a scale of 1-10) – as an alternative measure for patient satisfaction. Results are similar for each patient satisfaction measure, which is not surprising given recommend and rating have a correlation equal to 0.9.

Table 8. Sensitivity Analysis: Patient Satisfaction Measure

|  | Recommend | Rating |
| --- | --- | --- |
| Total margin | 0.0061  (0.0039) | 0.0062  (0.0039) |
| Mortality | -0.0568  (0.0393) | -0.0556  (0.0395) |
| Readmissions | 0.0928  (0.0787) | 0.0917  (0.0786) |
| Patient satisfaction | 0.0097*  (0.0052) | 0.0090*  (0.0049) |
| Uncompensated care | 0.2934  (0.4662) | 0.2590  (0.4690) |
| N | 882 | 882 |

Notes: Models are estimated using hospital fixed effects. Standard errors clustered at hospital level in parenthesis. All regressions include hospital characteristics and an indicator for year. Significance: ***p<0.01; **p<0.05; *p<0.1

Since our final analysis sample had larger hospitals and fewer hospitals in rural areas, we compared our results across underlying samples. Financial performance is statistically significant across all samples and regression specifications, and the coefficient is relatively stable. In models that include quality and patient satisfaction measures, the results are similar.

Table 9 presents results from both random effects and hospital fixed effects estimation.

Table 9. Panel Data Estimation Results

|  | (1) | (2) | (3) | (4) |
| --- | --- | --- | --- | --- |
| Total margin | 0.0090***  (0.0030) | 0.0061  (0.0039) | 0.0085**  (0.0042) | 0.0074  (0.0066) |
| Mortality | -0.0351  (0.0241) | -0.0568  (0.0393) | -0.0315  (0.0278) | -0.0286  (0.0382) |
| Readmissions | 0.0460  (0.0336) | 0.0928  (0.0787) | 0.0820  (0.0511) | -0.1075  (0.0911) |
| Patient satisfaction | 0.0030  (0.0024) | 0.0097*  (0.0052) | -0.0004  (0.0037) | -0.0014  (0.0049) |
| Uncompensated care | -0.7260  (0.4840) | 0.2934  (0.4662) | -0.5850  (0.4950) | -0.0605  (0.4554) |
| Estimation type | RE | FE | RE | FE |
| Same CEO both years? | No | No | Yes | Yes |
| N | 882 | | 330 | |
| Hausman test stat | 57.60*** | | 34.43*** | |

Notes: Standard errors clustered at hospital level in parenthesis. All regressions include hospital characteristics and year indicator. Significance: ***p<0.01; **p<0.05; *p<0.1

One of the findings from Joynt et al was that financial performance measures were not significantly correlated with hospital CEO compensation.[4] Their models included four financial performance measures: liquidity, capitalization, total margin, and occupancy rate. We only included total margin in our model since it measures of overall profitability. Capitalization provides an indicator for the long-run borrowing capacity for a hospital, and liquidity measures a hospital’s ability to pay its cash obligations. While these two financial measures might provide proxies for whether a hospital is at risk of bankruptcy or slow future growth, it is more likely that hospitals set targets for these measures based on organizational goals, and unlikely CEOs are judged on these metrics.

Table 10. Additional Financial Performance Measures

| Total margin | 0.0061  (0.0039) |
| --- | --- |
| Liquidity | -0.0568  (0.0393) |
| Capitalization | 0.0928  (0.0787) |
| Mortality | 0.0097*  (0.0052) |
| Readmissions | 0.2934  (0.4662) |
| Recommend | 0.0061  (0.0039) |
| Uncompensated care | -0.0568  (0.0393) |
| N | 882 |

Notes: Models estimated using hospital fixed effects. Standard errors clustered at hospital level in parenthesis. All regressions include hospital characteristics and indicators for year. Significance: ***p<0.01; **p<0.05; *p<0.1

Finally, we performed an analysis without CEO or hospital fixed effects (but with state fixed effects) across the unbalanced panel, balanced panel, and same CEO samples. Key results are shown below.

Table 11. Effect of Hospital Performance Measures, Additional Regressions

|  | (1) | (2) | (3) |
| --- | --- | --- | --- |
| Total Margin | 0.0101***  (0.0030) | 0.0072*  (0.0039) | 0.0074  (0.0058) |
| Mortality | -0.0071  (0.0222) | 0.0148  (0.0284) | -0.0046  (0.0383) |
| 30-day Readmissions | -0.0018  (0.0279) | 0.0267  (0.0393) | 0.1332*  (0.0686) |
| Recommend | 0.0024  (0.0020) | 0.0069**  (0.0032) | 0.0019  (0.0052) |
| Uncompensated Care | -1.064**  (0.4312) | -1.218**  (0.5930) | -2.223***  (0.7181) |
| Geographic controls | State | State | State |
| Sample | Unbalanced Panel | Balanced Panel | Balanced Panel |
| Estimation | OLS | OLS | OLS |
| Same CEO both years? | No | No | Yes |
| N | 1,664 | 882 | 330 |

References

1. Center to Advance Community Health & Equity. Community Benefit Insight [Internet]. [cited 2018 Feb 1]. Available from: http://www.communitybenefitinsight.org/?page=info.data_sources

2. Song PH, Lee S-YD, Toth M, Singh SR, Young GJ. Gender Differences in Hospital CEO Compensation: A National Investigation of Not-for-Profit Hospitals. Medical Care Research and Review. 2019 Dec;76(6):830–46.

3. Mulligan K, Choksy S, Ishitani C, Romley JA. New Evidence on the Compensation of Chief Executive Officers at Nonprofit U.S. Hospitals. Medical Care Research and Review. 2019 May 22;107755871984935.

4. Joynt KE, Le ST, Orav EJ, Jha AK. Compensation of Chief Executive Officers at Nonprofit US Hospitals. JAMA Internal Medicine. 2014 Jan 1;174(1):61.

1. A threshold of two decimal places was used since latitude and longitude in the AHA data varied at the third decimal place for hospitals over time. Distances between potential matches greater than 0.25 miles were checked manually. [↑](#footnote-ref-1)
2. Our method diverges slightly from CACHE’s since CACHE uses exact name matches while we allow for similar name matches. [↑](#footnote-ref-2)
3. Similarity scores were generated using the Excel Fuzzy Lookup add-in, which is based on a fuzzy matching algorithm. See <https://www.microsoft.com/en-us/download/details.aspx?id=15011> and <https://msdn.microsoft.com/en-us/library/ms345128.aspx> for additional details. [↑](#footnote-ref-3)
